# Molecular mechanisms and genetic features of cholangiocarcinoma: implications for targeted therapeutic strategies

**DOI:** 10.1093/pcmedi/pbaf021

**Published:** 2025-08-20

**Authors:** Xiao Lu, Shoujia Xu, Zhe Deng, Min-Jun Wang, Fei Chen

**Affiliations:** Department of Cell Biology, Naval Medical University, Shanghai 200433, China; Department of Cell Biology, Naval Medical University, Shanghai 200433, China; School of Medicine, Johns Hopkins University, Baltimore, MD 21218, USA; Department of Cell Biology, Naval Medical University, Shanghai 200433, China; Department of Cell Biology, Naval Medical University, Shanghai 200433, China

**Keywords:** cholangiocarcinoma, molecular mechanisms, molecularly targeted therapy, precision medicine

## Abstract

Cholangiocarcinoma (CCA) is a biologically diverse and highly aggressive cancer that arises from the biliary epithelium. It is typically divided into intrahepatic, perihilar, and distal types, each with distinct clinical behavior, genetic alterations, and therapeutic responses. Worldwide, the global incidence of CCA has risen steadily, accounting for nearly 15% of liver cancers and ∼3% of all gastrointestinal malignancies. CCA often presents at an advanced stage due to its silent onset and shows poor responsiveness to conventional chemotherapy, resulting in high mortality, accounting for ∼2% of cancer-related deaths worldwide. Risk factors include parasitic infections like liver flukes and chronic biliary diseases such as cholelithiasis and primary sclerosing cholangitis, although most cases have unknown origins. While early-stage patients may benefit from surgical resection or liver transplantation, these options are often not viable in advanced disease due to high relapse rates. In cases of unresectable or metastatic CCA, treatment remains difficult due to resistance and a lack of effective targeted therapies. This review systematically integrates the genomic, epigenetic, and signaling network mechanisms underlying CCA with their translational implications, providing a critical synthesis of the rapidly evolving field of targeted therapies, including recently approved Food and Drug Administration treatments and emerging novel agents. We specifically emphasize the key mechanisms of therapeutic resistance and corresponding strategies to overcome them, present an updated evaluation of vulnerabilities across distinct molecular subgroups, and explore the major challenges and future trajectories for advancing biomarker-driven precision medicine in CCA, thereby offering a forward-looking and clinically relevant perspective.

## Introduction

**Figure 1.**
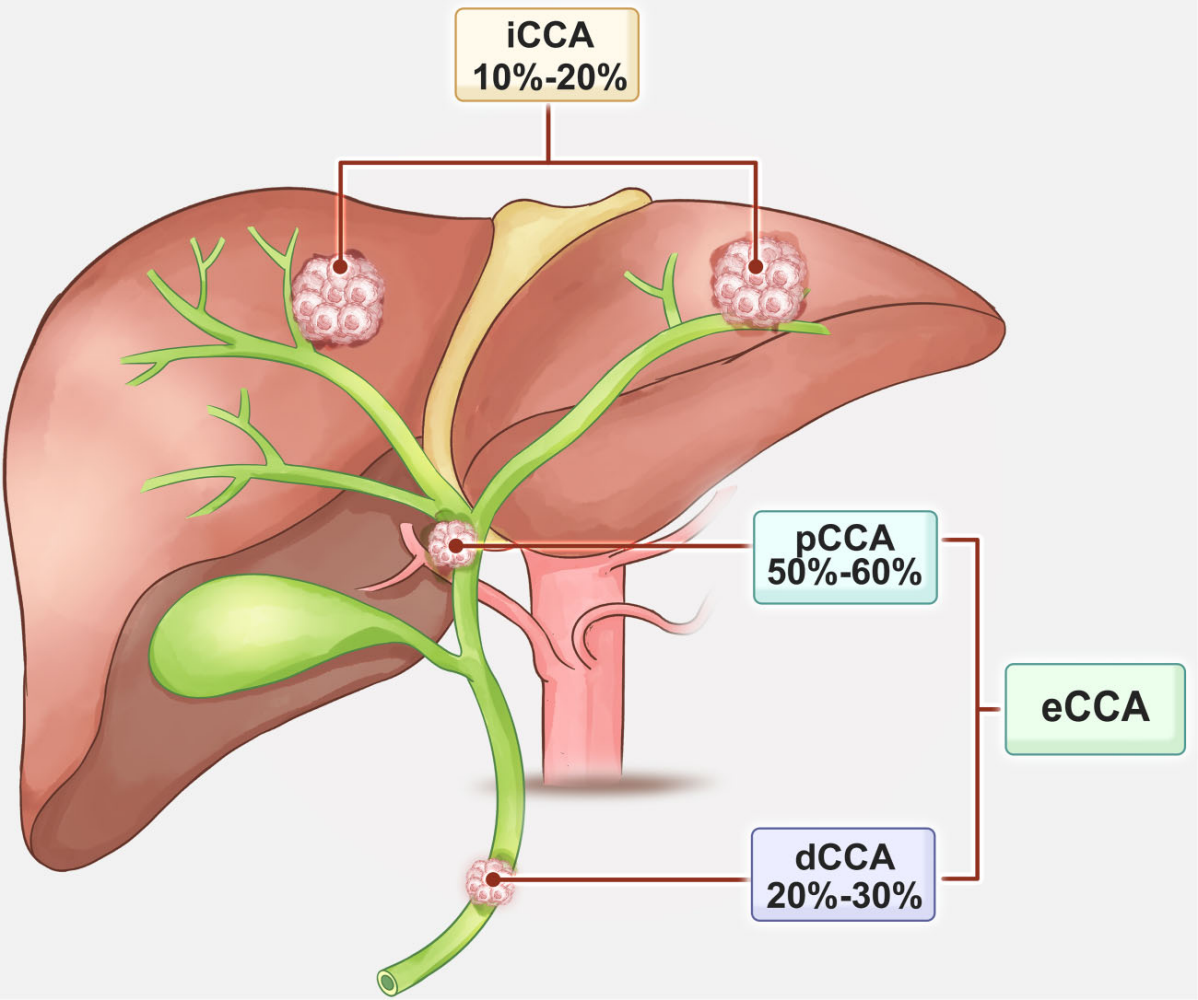
Subtypes of CCA. Based on anatomical origin, CCAs are broadly classified into iCCA, pCCA, and dCCA subtypes, and the corresponding proportions are shown.

Cholangiocarcinoma (CCA) is a highly aggressive malignancy derived from epithelial cells and can arise throughout the biliary tract, including regions within the liver parenchyma. Characteristically, CCA exhibits features of cholangiocyte differentiation and is thought to originate primarily from the biliary epithelial cells known as cholangiocytes. Nonetheless, alternative cells such as those from peribiliary glands or hepatocytes may also serve as the cell of origin, depending on the tumor's anatomical location and the nature of pre-existing liver disease [1, 2]. Based on anatomical origin, CCAs are broadly divided into intrahepatic (iCCA), perihilar (pCCA), and distal (dCCA) subtypes [3–5] (Fig. 1). pCCA and dCCA are often collectively referred to as extrahepatic CCA (eCCA). iCCA arises within the liver proximal to the second-order bile ducts, pCCA is situated between the second-order ducts and the point where the cystic duct enters the common bile duct, while dCCA originates below this insertion site within the common bile duct. The actual incidence rates of iCCA and pCCA remain uncertain due to the frequent misclassification of pCCA as iCCA in various national cancer registries [3, 6]. Moreover, advancements in diagnostic tools have improved the ability to distinguish iCCA from cancers of unknown primary origin, contributing to the apparent rise in iCCA incidence in recent decades. Each CCA subtype harbors distinct genetic mutations, clinical manifestations, and therapeutic implications [7]. While adenocarcinoma is the predominant histological type, rare variants such as adenosquamous and clear cell carcinomas have also been reported [8].

The global distribution of CCA displays marked geographic variability. In certain regions, parasitic infections caused by trematodes—flatworms commonly known as flukes—are a prominent etiological factor. Notably, in Southeast Asia, the liver fluke *Opisthorchis viverrini* is recognized as the primary driver of CCA [9]. Fluke-associated CCA can develop throughout the biliary system and may manifest as any of the three anatomical subtypes. Although these fluke-induced tumors may exhibit distinct pathogenic mechanisms, including unique genetic alterations, their clinical diagnosis and therapeutic management remain consistent with those of non-fluke-related CCA. In contrast, in Western countries, most CCA cases occur in the absence of identifiable risk factors, with the exception of a subset of patients diagnosed with primary sclerosing cholangitis [6, 7]. A more comprehensive understanding of the epidemiology, underlying risk factors, and molecular biology of CCA is essential for improving both preventive strategies and therapeutic outcomes.

In this review, we discuss the epidemiological features and risk factors of both liver fluke-associated and non-fluke-associated CCA, highlighting the roles of genomic, epigenetic, and cell signaling-related molecular drivers in CCA pathogenesis, as well as the development of molecularly targeted therapeutic strategies based on these insights.

## Epidemiology and risk factors

Based on data from the World Health Organization and the Pan American Health Organization covering 32 selected regions across Europe, the Americas, Asia, and Oceania, global mortality from CCA has shown a consistent upward trend over the past two decades [10–12].

Contemporary epidemiological studies have confirmed the persistent disparities in CCA outcomes across demographic and geographic dimensions. Mortality rates remain consistently higher in males than in females, with Asian populations continuing to demonstrate significantly elevated rates compared to their Western counterparts [13]. Notably, Japan reports the highest recorded male mortality (2.81/100 000) among Asian nations. In the United States, rising mortality trends observed over the past few years have revealed significant racial disparities: African Americans experienced the steepest increase (45%), followed by Asians (22%), and Whites (20%) [12, 14]. Age-standardized global incidence data further highlight extreme geographical variation—from hyperendemic regions like Northeast Thailand (85/100 000) to low-risk areas such as Canada (0.4/100 000) [15, 16]. These pronounced differentials are attributable to region-specific environmental exposures and potential genetic susceptibilities [6].

In parallel with incidence patterns, the global mortality of iCCA and eCCA demonstrates distinct epidemiological trajectories. Over recent decades, age-standardized incidence of iCCA has increased progressively in most regions worldwide, whereas eCCA incidence has generally declined [6, 7]. Among Western European nations, countries such as Ireland, the United Kingdom, Portugal, and Spain report the highest iCCA mortality rates in males, each exceeding 2 per 100 000 person-years. These nations also exhibit elevated female iCCA mortality, with Ireland recording the highest female rate on the continent at 2.66 per 100 000. Over the past decade, the most pronounced increases in iCCA mortality have been observed in the Baltic states. Latvia and Lithuania, in particular, show substantial average annual percentage changes (AAPC) in both sexes—18.14% and 23.74% in males, and 64.69% and 18.87% in females, respectively [10]. In the United States, iCCA is more frequently diagnosed in individuals ≥45 years old compared to younger populations, and its incidence is also higher among Hispanic individuals than non-Hispanics. Both groups are associated with poorer 5-year survival outcomes [17]. In addition, overall survival (OS) has been found to be lowest in African American patients, followed by American Indian and Alaska Native populations [17, 18].

Overall, eCCA exhibits lower incidence rates than iCCA across most countries [10]. Within Europe, only a few centrally located countries—namely Hungary, Germany, and Austria—report male eCCA mortality rates >1 per 100 000 person-years. Among females, Hungary stands out as the sole country where eCCA mortality surpasses this threshold. The most notable increases in eCCA mortality over the past decade have occurred in Hungary (32.96% in males; 25.92% in females), followed by Norway (32.02% in males; 27.39% in females), and Spain (21.86% in males; 26.15% in females) [10].

CCA has been associated with a variety of risk factors, both common and rare, as summarized in Table 1. While some of these factors are shared across all CCA subtypes, others appear to be subtype-specific and exhibit regional variation in their relevance. A shared feature among many of these risk elements is their link to persistent inflammation of the biliary epithelium and bile stasis [6]. Notably, several recognized risk factors—such as alcohol abuse, tobacco use, and viral infections including hepatitis B and hepatitis C—have shown global increases from 1990 to 2016 and may be contributing to rising CCA incidence [19]. Additionally, the worldwide prevalence of obesity, metabolic syndrome, and nonalcoholic fatty liver disease highlights emerging metabolic conditions that warrant further attention as potential contributors to CCA [20]. Despite these known associations, the majority of CCA cases in many regions remain sporadic, lacking identifiable etiologic factors.

**Table 1.** Risk factors for CCA^a^.

Risk type	Risk factor	Data source	OR or RR for iCCA	OR or RR for eCCA
Factors related to disease	Choledochal cyst [19]	Meta-analysis	OR 26.71	OR 34.94
	Choledocholithiasis [19]	Meta-analysis	OR 10.08	OR 18.58
	Cholelithiasis [19]	Meta-analysis	OR 3.38	OR 5.92
	Cholecystolithiasis [19]	Meta-analysis	OR 1.75	OR 2.94
	Cirrhosis [19]	Meta-analysis	OR 15.32	OR 3.82
	Chronic hepatitis B [19]	Meta-analysis	OR 4.57	OR 2.11
	Chronic hepatitis C [19]	Meta-analysis	OR 4.28	OR 1.98
	Inflammatory bowel disease [19]	Meta-analysis	OR 2.68	OR 2.37
	Obesity [19]	Meta-analysis	OR 1.14	OR 1.2
	Hypertension [19]	Meta-analysis	OR 1.10	OR 1.21
	Caroli disease [21]	Population-based study	OR 38	OR 97
	Primary sclerosing cholangitis [21]	Population-based study	OR 22	OR 41
	Hemochromatosis [21]	Population-based study	OR 2.1	No data
	Chronic pancreatitis [21]	Population-based study	OR 2.7	OR 6.6
	Type 2 diabetes mellitus [22]	Meta-analysis	OR 1.73	OR 1.5
	Nonalcoholic fatty liver disease [23]	Meta-analysis	OR 2.2	OR 1.5
	Metabolic syndrome [24]	Cohort study	RR 1.2	RR 1.2
Liver fluke	Liver fluke [25] (*Opisthorchis viverrini, Clonorchis sinensis*)	Meta-analysis	OR 5	No data
Unhealthy habit	Alcohol consumption [19]	Meta-analysis	OR 3.15	OR 1.75
	Cigarette smoking [19]	Meta-analysis	OR 1.25	OR 1.69
	1,2-Dichloropropane [26]	Retrospective study	RR 15	No data
	Asbestos [27]	Case control study	OR 4.8	OR 2.1
	Asbestos [20]	Case control study	OR 1.1–1.7	No association with eCCA

^a^eCCA, extrahepatic cholangiocarcinoma; iCCA, intrahepatic cholangiocarcinoma; OR, odds ratio; RR, relative risk.

Ongoing research is exploring the preventive potential of commonly used medications. Notably, aspirin [28] and statins [29] have been studied for their potential protective effects. Interestingly, aspirin use after CCA diagnosis has been correlated with a reduced mortality risk [30]. Furthermore, genetic polymorphisms in host genes involved in xenobiotic metabolism, DNA repair, immune modulation, folate metabolism, and multidrug resistance pathways have been implicated in susceptibility to CCA [6]. Although no genome-wide association studies have yet been published for CCA, a sufficiently powered genome-wide association study is highly anticipated.

## Molecular mechanisms of CCA

While multiple risk factors have been linked to the development of CCA, uncovering the definitive molecular drivers of its carcinogenesis remains critical for the advancement of effective treatment strategies. Genetic mutations have been identified in CCA, with driver gene alterations varying by anatomical subtype, ethnicity, and underlying causes [31, 32]. Nonetheless, these genetic changes alone do not fully explain the extensive clinical heterogeneity and transcriptomic complexity observed in CCA. Therefore, epigenetic modifications and the dynamic interactions within the tumor microenvironment are increasingly recognized as important contributors to CCA pathobiology [33]. Furthermore, both genetic and epigenetic alterations can impact protein function, potentially disrupting cellular signaling pathways and molecular networks, thereby facilitating CCA progression [34].

### Genetic mechanisms in CCA pathogenesis

#### iCCA

Extensive genomic studies on iCCA have uncovered a wide spectrum of driver mutations. These genetic alterations commonly impact crucial signaling pathways, notably those involved in chromatin remodeling, metabolism, and cell cycle control. Significant mutations have been reported in genes including BAP1, ARID1A, IDH1/2, PBRM1, and FGFR2. Approximately 47% of iCCA cases present alterations in at least one gene associated with chromatin remodeling [35]. Somatic mutations in BAP1, which encodes a nuclear deubiquitinase, occur in 9% to 25% of iCCA. ARID1A, a component of the SWI/SNF chromatin-remodeling complex, shows mutations in ∼19% to 22% of cases. Missense mutations in IDH1 and IDH2 are found in 15% to 20% of iCCA, though they are less frequent in Asian iCCA patients and in pCCA or dCCA subtypes [32, 36]. Isocitrate dehydrogenase (IDH) mutations disrupt hepatocyte differentiation by generating 2-hydroxyglutarate and downregulating hepatocyte nuclear factor 4α (HNF-4α), a key factor for hepatocyte identity and quiescence. Inhibiting HNF-4α suppresses hepatocyte differentiation and promotes proliferation, contributing to premalignant biliary lesions and eventual metastatic iCCA [37]. Multi-omics profiling has identified an IDH-mutant-enriched iCCA subtype, characterized by high mitochondrial activity and low chromatin modifier expression, including epigenetic silencing of ARID1A, suggesting its oncogenic significance [38]. Additionally, a recent study by Bardeesy's team constructed a molecular and functional atlas of 63 biliary tract cancer (BTC) cell lines. By integrating multi-omics data and genome-wide CRISPR screening, the study identified key subtype-specific dependencies such as EGFR signaling (linked to 3p loss), SHP2 in FGFR2 fusion tumors, and MAPK pathway components in BRAF V600E-mutant cells. Transcriptomic clustering revealed two BTC subtypes—bi-lineage and ductal—with distinct molecular features and prognoses. This atlas provides a valuable resource for understanding BTC heterogeneity and supports the development of subtype-guided therapeutic strategies [39].

**Figure 2.**
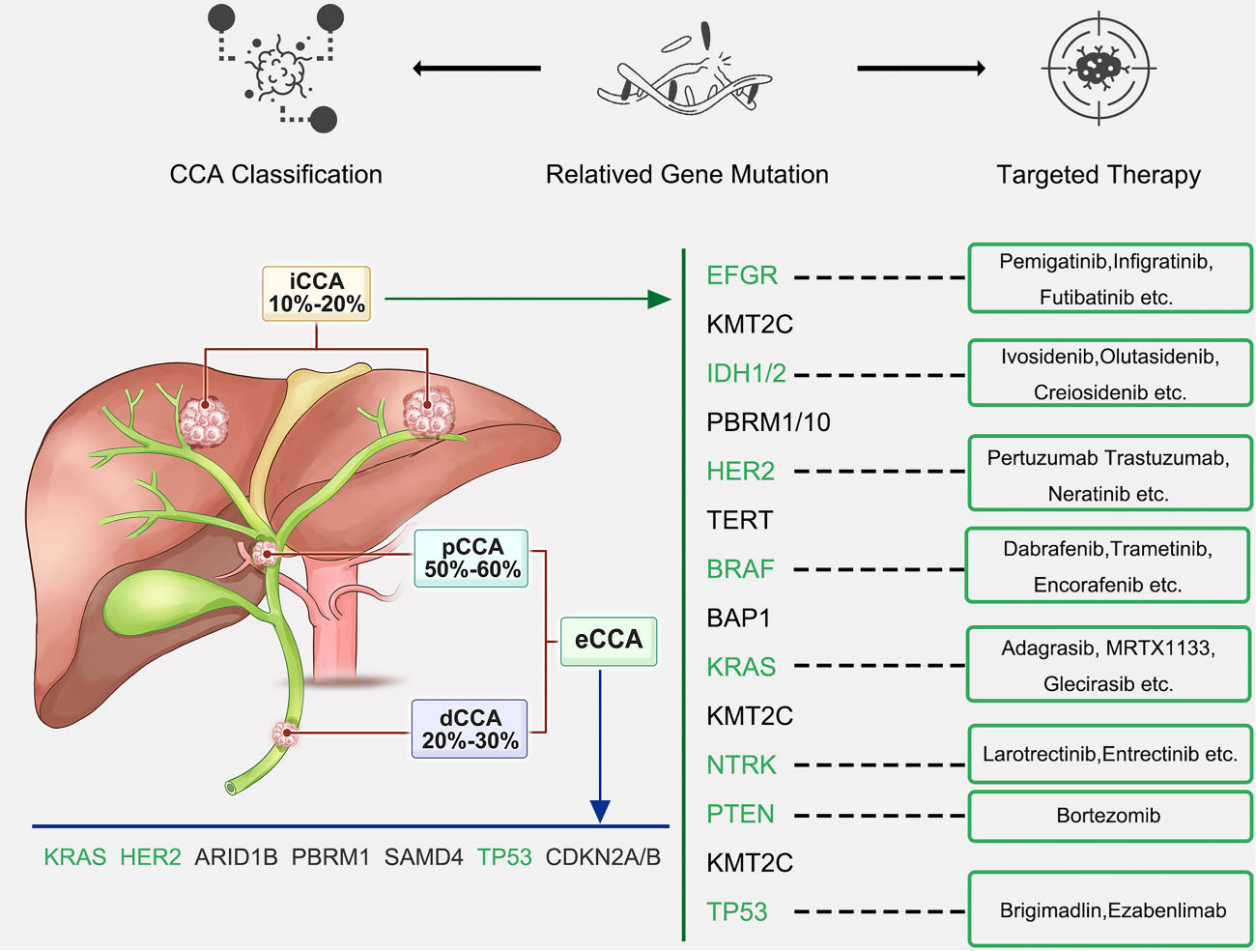
Genetic alterations and corresponding targeted therapies across different CCA subtypes. Commonly mutated genes in iCCA include IDH1/2, BAP1, ARID1A, and FGFR2, among others, while eCCA frequently harbors mutations in KRAS, HER2, TP53, and CDKN2A/B. Representative genetic alterations across CCA subtypes and their corresponding targeted therapies are summarized, including EGFR inhibitors (pemigatinib, infigratinib, futibatinib), IDH1/2 inhibitors (ivosidenib, olutasidenib, crelosidenib), HER2-targeted agents (pertuzumab, trastuzumab, neratinib), BRAF inhibitors (dabrafenib, trametinib, encorafenib), KRAS inhibitors (adagrasib, MRTX1133, glecirasib), NTRK inhibitors (larotrectinib, entrectinib), and other agents such as bortezomib (targeting PTEN), brigimadlin, and ezabenlimab (targeting TP53).

Mutations in PBRM1, another ATP-dependent chromatin-remodeling complex subunit, are found in 6% to 17% of iCCA cases [35, 40]. Additionally, Zou *et al*. identified iCCA-specific somatic mutation signatures and reported several candidate driver genes such as TP53, KRAS, IDH1, PTEN, ARID1A, EPPK1, ECE2, and FYN [40]. A study by Chaisaingmongkol *et al*. identified 22 potential driver genes, including APC, KMT2C, GNAS, KRPB1, ADAMTS20, ERBB3, ERBB2, ATM, KAT6B, MAP2K4, and RNF43, and found that eight genes (TP53, ARID1A, ARID2, CSMD3, RYR2, NF1, PRKDC, and PSIP1) are shared between iCCA and hepatocellular carcinoma (HCC), indicating potential overlap in their molecular pathways [32]. In addition to point mutations, gene fusions involving FGFR, especially FGFR2, have been detected in 10% to 15% of iCCA cases, but are rarely seen in eCCA [41]. These FGFR2 fusions have diverse fusion partners, leading to receptor activation [41] (Fig. 2).

#### eCCA

iCCA and eCCA demonstrate distinct molecular mutation profiles. Genetic alterations such as KRAS, TP53, SMAD4, and STK11 are observed more frequently in eCCA [42, 43]. In a recent comparative study, Zhang *et al*. investigated the genetic differences between iCCA and pCCA, identifying mutations enriched in pCCA, including RBM10, TGFBR2, PIK3R1, ELF3, NACC1, and METTL14 [44]. Notably, they emphasized the role of post-transcriptional modification-associated genes such as METTL14 and RBM10 as potential drivers in pCCA [44]. In addition, some driver mutations are shared between iCCA and eCCA, including ERBB2 (erb-b2 receptor tyrosine kinase 2) amplification and mutations in RASA1, MAP2K4, and SF3B1 [45] (Fig. 2).

### Epigenetic mechanisms in CCA pathogenesis

**Figure 3.**
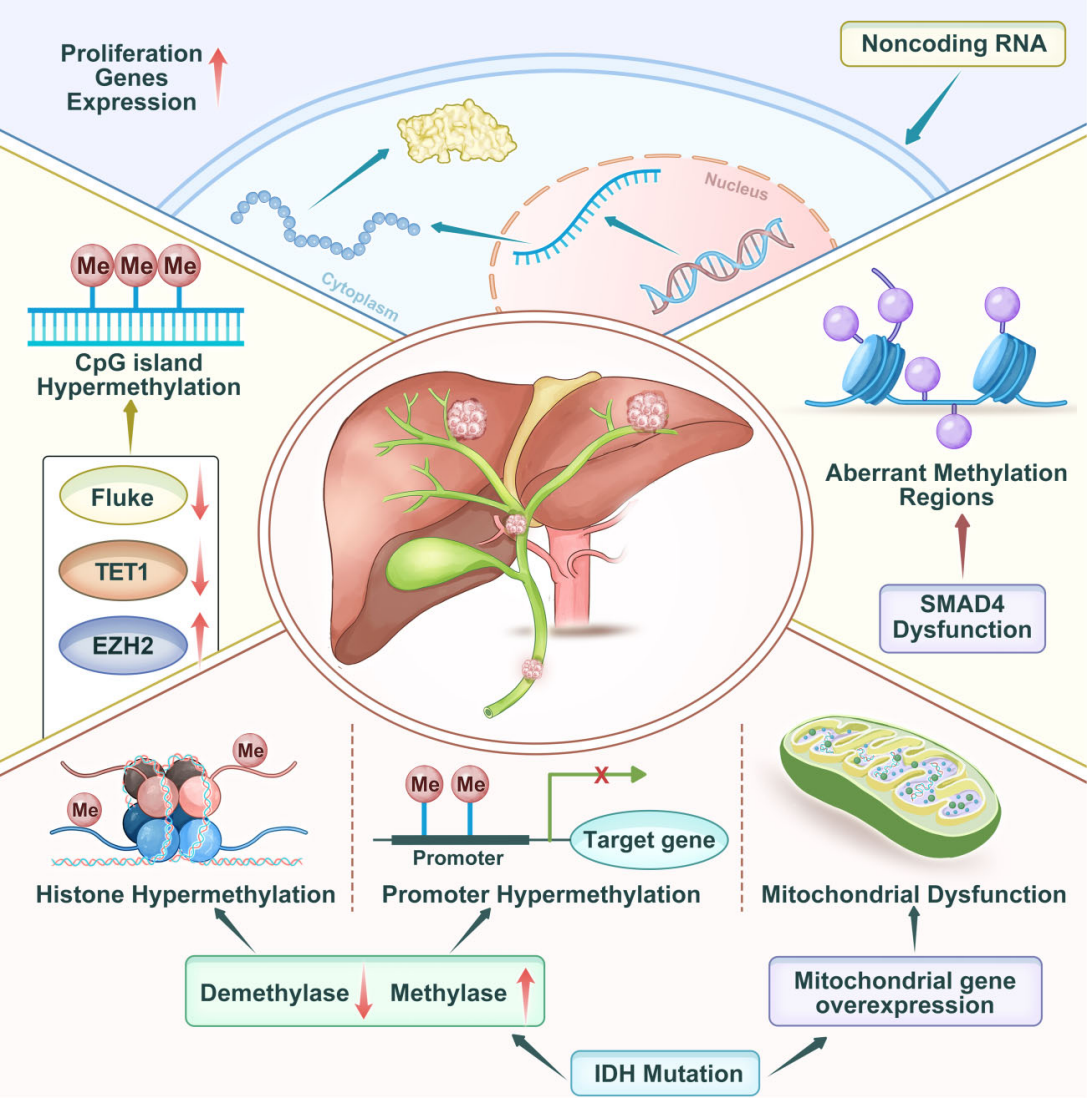
Epigenetic alterations in CCA. Dysregulation of histone modifications, DNA methylation, and non-coding RNAs cooperatively promote CCA tumorigenesis through target-gene regulation. CpG island hypermethylation—induced by factors such as liver fluke infection, altered TET1 activity, and EZH2 overexpression—silences tumor suppressor genes. Histone hypermethylation, regulated by the imbalance between methylases and demethylases, modifies chromatin accessibility and gene transcription. Promoter hypermethylation directly represses target gene expression. IDH mutations alter the cellular methylation landscape and contribute to promoter hypermethylation. Aberrant methylation in specific chromatin regions is associated with SMAD4 dysfunction, impairing transforming growth factor-β signaling. Non-coding RNAs modulate transcriptional and post-transcriptional gene regulation. Mitochondrial dysfunction, often linked to mitochondrial gene overexpression, further disrupts cellular homeostasis and promotes malignant transformation.

Epigenetic mechanisms are increasingly recognized as crucial contributors to the initiation and progression of CCA, influencing tumor behavior without altering the DNA sequence [46]. Dyregulations in DNA methylation, histone modification patterns, and the expression of non-coding RNAs can lead to transcriptional and gene expression imbalances, undermining cellular equilibrium and fostering malignant transformation (Fig. 3). In this section, we provide an overview of pivotal studies that investigate how epigenetic alterations serve as key drivers in the pathogenesis of CCA.

#### Aberrant DNA methylation

Accumulating evidence indicates that methylation patterns are dysregulated in CCA cells relative to normal biliary epithelial cells [47], with widespread hypermethylation observed across numerous CpG sites in CCA [48, 49]. Integrative analyses combining DNA sequencing, transcriptomics, and DNA methylation profiling have delineated four molecular subgroups of CCA with distinct clinical outcomes. Among them, two subgroups characterized by prominent CpG island hypermethylation were particularly notable [48]. These hypermethylated clusters showed strong correlations with liver fluke-associated tumors, elevated mutation frequencies, suppressed expression of the DNA demethylase TET1, increased expression of the histone methyltransferase EZH2, and enhanced rates of cytosine deamination events [49].

**Figure 4.**
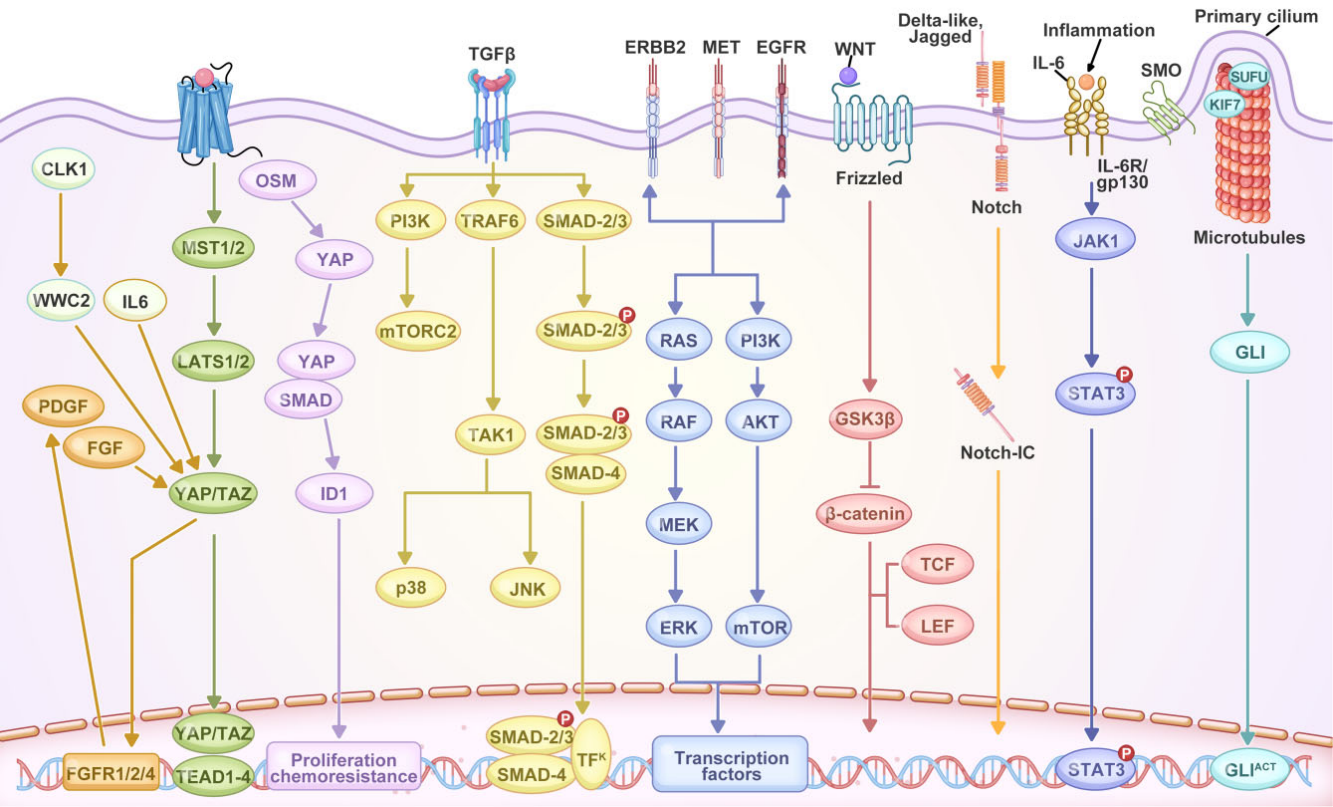
Major signaling pathways implicated in CCA pathogenesis. The Hippo pathway (WWC2–MST1/2–LATS1/2–YAP/TAZ) modulates proliferation and chemoresistance via TEAD- and FGFR-mediated transcription. TGF-β signaling activates SMAD2/3–SMAD4 and non-canonical p38/JNK branches to regulate transcriptional programs. ERBB2, MET, and EGFR drive RAS–RAF–MEK–ERK and PI3K–AKT–mTOR pathways, promoting growth and survival. Wnt signaling inhibits GSK3β, leading to β-catenin activation of TCF/LEF target genes. Notch activation produces Notch intracellular domain (Notch-IC), which regulates nuclear transcription. Interleukin-6/JAK1–STAT3 signaling links inflammation to oncogenic transcription, while Hedgehog/SMO–GLI promotes GLI-dependent gene expression. Crosstalk among these cascades supports tumor progression and therapy resistance.

In addition to TET1 and EZH2, the loss of Smad4 function has been shown to significantly influence DNA methylation landscapes in CCA [50]. In an albumin-Cre-driven murine model with hepatic expression of mutant KRAS, TP53, and SMAD4, reduced representation bisulfite sequencing revealed that Smad4-regulated hypomethylated regions tended to be located farther from transcriptional start sites compared to other genomic regions. This finding indicates that Smad4 may exert a distinct regulatory effect on distal DNA methylation. Further comparisons of cell lines derived from mice with either intact or deleted Smad4 showed differential methylation patterns across genomic elements, including promoters, exons, introns, intergenic regions, and transcriptional start sites. Notably, loss of Smad4 resulted in widespread hypomethylation, particularly in intergenic regions, implying that Smad4 contributes to the regulation of genome-wide methylation architecture in CCA [50]. Although the precise molecular mechanisms remain to be fully elucidated, it is still uncertain whether Smad4 directly interacts with the DNA methylation machinery. Alternatively, Smad4 may exert its tumor-suppressive effects indirectly through dysregulation of cell cycle control or aberrant proliferative pathways. While previous studies have demonstrated that transforming growth factor-β (TGF-β)/SMAD4 signaling modulates epigenetic regulation of specific genes like RUNX1T1 and VAV1, these findings suggest that SMAD4 may also mediate broader epigenetic alterations at the genome level in CCA (Fig. 4).

IDH1 and IDH2 enzymes play crucial roles in cellular metabolism as two of the three isoforms of isocitrate dehydrogenase. These enzymes catalyze the oxidative decarboxylation of isocitrate to yield α-ketoglutarate and CO₂. As noted in the section on genetic drivers, mutations in the IDH1 and IDH2 genes are present in CCA as well as in other malignancies [38, 51]. These mutations result in the accumulation of the oncometabolite 2-hydroxyglutarate, which inhibits the activity of histone and DNA demethylases. This inhibition leads to widespread hypermethylation of DNA and histones, thereby inducing the overexpression of genes encoding enzymes of the tricarboxylic acid cycle, mitochondrial ribosomal proteins, components of the electron transport chain, and proteins involved in mitochondrial architecture. Such alterations cause significant disruption of oxidative phosphorylation and mitochondrial biogenesis [52]. IDH1 and IDH2 mutations are far more prevalent in CCA compared to pCCA or dCCA subtypes, and their characteristic impact on DNA methylation renders IDH-mutated tumors relatively easy to identify [53].

Numerous studies have implicated various elements of the Wnt signaling cascade in the pathogenesis of CCA [48], even though mutations in the CTNNB1/β-catenin gene are relatively rare in this cancer type. These observations indicate that activation of the Wnt pathway may either serve as a common initiating factor or represent a convergent mechanism in CCA development. Several Wnt-associated genes have been linked to this process. A genome-wide investigation into aberrant promoter methylation in human CCA specimens identified hypermethylation of genes involved not only in the Wnt pathway but also in the TGFβ and phosphatidylinositol 3-kinase pathways. Specifically, dysregulated methylation was observed in multiple Wnt-related genes, including SOX17, WNT3A, DKK2, SFRP1, SFRP2, and SFRP4 [48]. For example, SOX17 plays a critical role in cholangiocyte differentiation during embryonic development. Notably, its expression is suppressed in CCA due to hypermethylation of both its promoter and regulatory regions [49]. An inverse relationship has been observed between the extent of promoter and SOX17 expression levels. Reduced SOX17 expression in CCA is linked to poorer outcomes following surgical resection. Conversely, ectopic expression of SOX17 in CCA cells results in reduced proliferation, lower oxidative stress, and increased apoptosis. Furthermore, hypermethylation-induced silencing of the SOX17 gene has been associated with the activation of Wnt signaling, while re-expression of SOX17 inhibits Wnt3a-mediated cellular proliferation [49]. These findings suggest that therapeutic strategies aimed at restoring SOX17 expression may hold promise in the treatment of CCA.

#### Aberrant histone modification

Histone acetylation and deacetylation are dynamically regulated by the opposing actions of histone acetyltransferases and histone deacetylases (HDACs), which together play a crucial role in modifying chromatin structure and regulating gene expression [54]. Several studies have investigated the role of HDACs in CCA, though their findings are somewhat inconsistent. While one report indicated that HDAC1, HDAC2, and HDAC8 levels remain unchanged in CCA tissues, HDAC3 was found to be significantly overexpressed. Elevated HDAC3 expression has been linked to increased proliferation of CCA cells, suppression of p53-mediated apoptosis, and reduced patient survival [55]. In contrast, another study reported upregulated expression of HDAC2, HDAC3, and HDAC8 in CCA tissues, with high levels of HDAC2 and HDAC3 correlating with poor clinical outcomes [56]. Similarly, high HDAC3 expression has been consistently associated with poor prognosis in CCA. Notably, HDAC8 expression shows a negative correlation with the Ki67 index, and its reduced expression in iCCA is also linked to worse prognosis [57]. Mechanistically, HDAC3 may deacetylate cMyc at the K323 residue, enhancing its stability by preventing ubiquitin-mediated degradation. Stabilized cMyc can upregulate LDHA and PKM2, lowering intracellular pyruvate levels, which may, in turn, diminish HDAC3 inhibition and suppress CCA cell apoptosis [58]. Additionally, Sirt2 contributes to cMyc stabilization through deacetylation, and elevated Sirt2/cMyc expression promotes metabolic reprogramming, mitigates oxidative stress, enhances serine biosynthesis, and drives CCA cell proliferation by promoting PDHA1 phosphorylation [59]. Overall, although current evidence suggests a link between aberrant histone modifications and CCA progression, research in this area remains limited, and further mechanistic insights are needed.

#### Noncoding RNA

Recent studies have increasingly underscored the role of noncoding RNAs (ncRNAs)—including long noncoding RNAs (lncRNAs), microRNAs (miRNAs), and circular RNAs (circRNAs)—in the pathogenesis of CCA, its aggressive clinical behavior, and patient prognosis [60–62]. Transcriptomic profiling and assay for transposase-accessible chromatin sequencing in CCA cell lines have identified nuclear lncRNA LINC00313 as a regulator of the Wnt signaling pathway with oncogenic potential. Mechanistically, LINC00313 facilitates Wnt activation by interacting with ACTL6A and BRG1, which leads to transcriptional upregulation of TCF7 and SULF2, thereby promoting CCA progression in both cell culture and animal models [63].

Similarly, LOXL1-ASI, another lncRNA markedly upregulated in CCA tissues, modulates the JAK2-STAT3 pathway by affecting JAK2 ubiquitination and subsequent degradation [64]. In another example, lncRNA DLEU1 was shown to facilitate CCA progression by sponging miR-149–5p, which elevates YAP1 expression. YAP1 acts as a transcriptional coactivator and promotes SOX2 expression via the TEAD2 transcription factor, suggesting a DLEU1/miR-149–5p/YAP1/TEAD2/SOX2 signaling axis that contributes to CCA tumorigenesis [65]. Additionally, lncRNA LINC01812 has emerged as a critical regulator in the tumor microenvironment of CCA. Recent findings reveal that LINC01812 is significantly upregulated in tumor tissues and is selectively packaged into tumor-derived exosomes. These exosomes are internalized by macrophages, driving their polarization toward the M2 phenotype. The resulting M2 macrophages promote perineural invasion (PNI) of CCA cells, as demonstrated by coculture and neural infiltration assays. This lncRNA-driven exosomal communication highlights a novel mechanism by which CCA modulates immune cells to facilitate PNI, offering potential therapeutic targets focused on the LINC01812–M2 axis [66].

Various investigations have also revealed that circRNAs play crucial roles in CCA development by regulating gene expression through miRNA sponging, echoing the mechanism of DLEU1. For instance, Liu *et al*. demonstrated that circPCNXL2 is significantly upregulated in iCCA and promotes tumor progression by interacting with serine-threonine kinase receptor-associated protein, thereby activating the ERK/MAPK pathway. Moreover, circPCNXL2 can sponge miR-766–3p, leading to enhanced expression of SRSF1, further promoting iCCA growth [67].

An alternative mechanism was presented by Chen *et al*., who identified circRNA Circ_0 020 256 as being highly expressed in tumor-associated macrophage-derived exosomes in CCA. *In vitro* studies showed that Circ_0 020 256 promotes CCA progression by targeting intracellular miR-432–5p, resulting in increased E2F3 expression [68]. Additionally, multiple miRNAs have been found to modulate oncogenic or tumor-suppressive pathways, and their regulatory roles are often altered due to sponging by other ncRNAs, ultimately affecting cell proliferation and tumor progression. A recent study identified circ_0 084 927 as a key regulator in iCCA. It promotes tumor progression and gemcitabine resistance by sponging miR-4725–5p to upregulate PDPK1 and activate the AKT/mTOR pathway. Exosomal circ_0 084 927 also mediates drug resistance transfer, highlighting its potential as a prognostic and therapeutic target [69].

miRNAs have been more extensively studied compared with lncRNAs and circRNAs. A growing body of evidence supports the idea that miRNAs can serve as both promotors and suppressors of CCA pathogenesis [70, 71]. Initial studies revealed that certain miRNAs are overexpressed in various CCA cell lines, with some miRNAs contributing to gemcitabine resistance and enhancing cellular proliferation *in vitro* [72]. These miRNAs have been shown to inhibit programmed cell death and interfere with p53-associated signaling, while simultaneously promoting Wnt/β-catenin pathway activation [73–75]. Moreover, miRNAs exert distinct influences on the epithelial–mesenchymal transition (EMT) process. For example, miR-200c has been found to suppress EMT, whereas miR-21 facilitates EMT in CCA cell models [76, 77]. In addition, miR-424–5p has been shown to inhibit the proliferation and metastatic potential of iCCA cells by targeting circACTN4/YAP1 expression and suppressing β-catenin signaling activation [78].

The evidence discussed above represents only a fraction of the existing research and serves to illustrate the diverse mechanisms by which ncRNAs can influence CCA cell behavior. While numerous studies have identified correlations between ncRNAs and aggressive CCA phenotypes, further comprehensive mechanistic investigations are necessary to establish ncRNAs as true drivers of CCA development. Among the various ncRNA classes, miRNAs have been the most extensively investigated in the context of CCA, with current findings underscoring their potential role as key contributors to CCA pathogenesis (Fig. 4).

### Molecular signaling networks in CCA pathogenesis

Cholangiocarcinogenesis results from a multifaceted interaction between extracellular ligands—such as pro-inflammatory cytokines, growth factors, and bile acids—within the tumor microenvironment, along with the overexpression or aberrant activation of cell surface receptors and disrupted intracellular signaling pathways. This intricate network ultimately drives cellular proliferation, survival, and both genetic and epigenetic alterations. Based on transcriptomic analyses and gene set enrichment approaches, iCCA has been stratified into two molecular subtypes: an ‘inflammation’ subtype (38%) and a ‘proliferation’ subtype (62%), each showing distinct pathway enrichment [34]. Tumors in the inflammation category display activation of immune-related signaling cascades. In contrast, the proliferation subtype is marked by enrichment in well-established oncogenic pathways, including aberrant receptor tyrosine kinase (RTK) signaling, RAS–RAF–ERK and PI3K–AKT–mTOR pathways, insulin-like growth factor receptor 1, MET, polo-like kinase 1, aurora kinase A, KRAS mutations, stem-like genomic characteristics, and focal deletions in the Hippo signaling pathway, particularly involving SAV1 [34, 79, 80].

Chronic inflammation and fibrosis contribute to cholangiocyte transformation through a multistep process, largely by providing extracellular ligands that influence various signaling pathways. A key example is how bile acids drive cholangiocarcinogenesis by activating EGFR, increasing the expression of COX2, MCL1, and interleukin-6 (IL-6), and reducing levels of the farnesoid X receptor (FXR) [81, 82]. Notably, FXR expression has been found to be diminished in human CCA tissues compared to adjacent non-tumorous liver, and this reduction is associated with tumor differentiation [83]. In contrast, the bile acid receptor TGR5 is upregulated in CCA specimens, with elevated expression correlating with poorer clinical outcomes, such as PNI [83]. Furthermore, CCA—particularly iCCA and pCCA subtypes—exhibits a desmoplastic stroma enriched with cancer-associated fibroblasts (CAFs). These CAFs engage in reciprocal communication with tumor cells via paracrine mediators, including heparin-binding EGF-like growth factor, stromal cell-derived factor 1 (SDF1), platelet-derived growth factor-B (PDGF-B), and various extracellular matrix components [84].

Activation of RTK signaling is a common molecular event observed across various subtypes of CCA. Notably, abnormal expression of RTKs such as EGFR, ERBB2, and MET has been detected in multiple CCA subgroups, and this dysregulation correlates with poorer clinical outcomes [34, 79]. RTK signaling primarily initiates downstream cascades, particularly the RAS–MAPK and PI3K–AKT–mTOR pathways. Importantly, activating mutations in KRAS contribute to RAS–MAPK pathway activation and are found ubiquitously across CCA subtypes, whereas BRAF mutations appear to be more specific to iCCA [85]. Of particular interest, oncogenic chromosomal rearrangements resulting in FGFR2 fusion genes are almost exclusively identified in iCCA cases [86–88]. These findings suggest that RTK-related signaling pathways harbor several targetable genetic alterations, offering promising opportunities for therapeutic intervention at different molecular levels.

Integrated microarray analyses have revealed that developmental signaling pathways such as Notch, WNT, and TGFβ are more prominently activated in iCCA than in HCC [89]. These pathways, typically involved in biliary development, become reactivated during liver injury and chronic inflammation—well-established risk factors for iCCA [90]. In ductular reactive cells, signaling cascades such as Notch, WNT, Hippo–YAP, and Hedgehog are triggered under these conditions. The Notch pathway, in particular, plays a critical role in biliary regeneration, cell proliferation, tubulogenesis, fibrosis, and the maintenance of the hepatic stem cell niche. Consequently, its activation has also been used as a predictor of poor prognosis in combined HCC–CCA [91]. Loss-of-function mutations in components like JAG1 or NOTCH2 are associated with impaired liver regeneration and Alagille syndrome [92], while heightened Notch activity has been linked to the development of primary liver cancers [93]. Aberrant or elevated expression of Notch receptors has been detected in both iCCA and eCCA, including pCCA and dCCA subtypes [94–96]. Functionally, activation of Notch signaling has been implicated in the transdifferentiation of hepatocytes into cholangiocytes during tumorigenesis [97–100]. Experimental studies have shown that overexpressing the intracellular domain of NOTCH1 in hepatocytes can lead to iCCA formation in murine models [97, 99, 100]. Conversely, suppression of NOTCH2—whose expression correlates with well-differentiated iCCA—significantly decreases tumor growth in various mouse models of liver cancer, including iCCA [98, 101]. Furthermore, NOTCH3 overexpression has been implicated in iCCA development and progression by enhancing cell survival through the PI3K–AKT pathway [102].

The WNT–β-catenin signaling pathway is frequently activated in the majority of CCA [103, 104], partly due to Wnt ligands secreted by inflammatory macrophages within the tumor stroma [105, 106], and also as a result of DNA methylation changes affecting this pathway [48] or mutations in genes encoding essential components of the canonical WNT–β-catenin cascade [107]. Notably, in CCA tumor tissues, the promoter of SOX17—a negative regulator of the WNT–β-catenin pathway—was found to be hypermethylated when compared with adjacent healthy tissue, and this methylation status correlated with a poorer prognosis following tumor resection [49]. Importantly, SOX17 has been identified as a key regulator of cholangiocyte differentiation and acts as a tumor suppressor in *in vitro* CCA models [49].

YAP, a transcriptional co-activator, is normally suppressed by the Hippo pathway through MST1 or MST2, but can also be activated through Hippo-independent mechanisms, including inflammatory cues and alterations in the ECM composition and stiffness [108]. Elevated nuclear localization of YAP has been observed in CCA tissue samples and is associated with poorer patient outcomes [109, 110]. *In vitro* and *in vivo* experiments using CCA cell lines have demonstrated that YAP activation can be triggered by IL-6, PDGF, fibroblast growth factor (FGF) and oncostain M (OSM) secreted by TAM [111–113]. PDGF and FGF contribute to a feed-forward loop that sustains YAP activity, with YAP in turn promoting the expression of genes from these pathways, such as FGFR1, FGFR2, and FGFR4 [111, 112]. OSM facilitates the nuclear translocation of YAP and its interaction with SMAD proteins, leading to the upregulation of inhibitor of DNA binding 1 (ID1) expression. This activation of the OSM–YAP–ID1 axis contributes to CCA progression and enhances chemoresistance [113]. A recent study highlights CLK1 as a critical driver of iCCA progression by synergizing with AKT to initiate tumor development and activating the Hippo–YAP signaling pathway. Mechanistically, CLK1 appears to regulate YAP activity via WWC2, indicating that inhibiting YAP may offer a promising therapeutic approach against CLK1-driven tumorigenesis [114]. Furthermore, AKR1C1–CYP1B1–cAMP signaling axis, AMDHD1/TGF-β signaling pathway, EZH2-mediated WNT7B/β-catenin pathway, VEGF/FGF signaling, MUC3-MAPK/ERK pathway, EGFR/SREBP-1 pathway, and lipopolysaccharide-mediated METTL3/PI3K/AKT signaling have been identified in CCA development [115–120]. For example, AKR1C1 suppresses ferroptosis by promoting CYP1B1 degradation and downregulating its transcription via AHR. This axis modulates ferroptosis sensitivity through the cAMP–PKA pathway, offering a potential therapeutic target for eCCA, especially in combination with ferroptosis inducers [115] (Fig. 4).

## Molecularly targeted therapies

A large-scale genomic analysis of tumor samples from 1 104 patients with advanced CCA identified TP53 (38.1%), CDKN2A/B (28.8%), KRAS (21.9%), ARID1A (15.7%), SMAD4 (11.3%), BAP1 (10.6%), IDH1 (10.5%), PBRM1 (10.0%), FGFR2 (9.4%), ERBB2 (7.6%), PIK3CA (7.0%), MDM2/FRS2 (5.8%), and BRAF (4.7%) as the most frequently altered genes [121]. These findings were further supported by a comprehensive genomic profiling study involving 3 634 CCA patients, which consistently reported similar mutation patterns [122]. Together, these studies define the mutational landscape of CCA and offer essential molecular insights that may guide future therapeutic approaches. We next discuss molecular targeted therapy strategies based on these recurrent gene mutations (Table 2 and Fig. 2).

**Table 2.** Summary of molecularly targeted therapies in CCA.

Target	Drug	Trial No.	Phase	Description	Outcome
FGFR	Pemigatinib	NCT02924376	Ⅱ	Multicenter, open-label, single-arm, multicohort study	ORR 37%, PFS 7 months, OS 17.5 months
		NCT04256980	Ⅱ	Multicenter, single-arm study	ORR 50%, PFS 6.3 months, OS 23.9 months
		NCT03656536	Ⅲ	Open-label, randomized, active-controlled, multicenter, global study	No study results posted as study is ongoing
	Infigratinib	NCT02150967	Ⅱ	Multicenter, open-label study	ORR 23.1%, PFS 7.3 months, OS 12.2 months
		NCT03773302	Ⅲ	Multicenter, open-label, randomized study	ORR 37.9%, PFS 7 0.4 months, OS 14.4 months
	Futibatinib	NCT02052778	Ⅱ	Single-arm study	ORR 41.7%, PFS 8.9 months, OS 20 months
		NCT04093362	Ⅲ	Multicenter, open-label, randomized study	No study results posted due to poor recruitment.
	Erdafitinib	NCT04083976	Ⅱ	International, open-label, single-arm study	ORR 52%, DCR 07%
		NCT02699606	Ⅱa	Open-label, single-arm study	ORR 40.9%, PFS 5.6 months, OS 40.2 months
	Tinengotinib	NCT04919642	Ⅱ	Open-label, multicenter phase study	PFS 5.2 months
		NCT05948475	Ⅲ	Open-label, multicenter study	No study results posted as study is ongoing
	Derazantinib	NCTO3230318	I/II	Multicenter, open-label study	ORR 20.7%, PFS 5.7 months
		NCT05174650	Ⅱ	Open-label, single-arm, multicenter study	No study results posted
	Gunagratinib	NCT03758664	I/IIa	Open-label, non-randomized, dose-escalation, dose-extension, first-in-man study	ORR 33.3%
IDH1	Ivosidenib	NCT02073994	I	Multicenter, open-label, dose-escalation and expansion study	PFS 3.8 months, OS 13.8 months
		NCT02989857	III	Multicenter, randomized, double-blind, placebo- controlled study	ORR 3.2%, PFS 2.7 months, OS 10.3 months
	Olutasidenib	NCT03684811	I/II	Part 1: single agent, open-label study. Part 2: open-label study in combination with other anti-cancer agents	PFS 8.3 months for single agent
	Crelosidenib	NCT04521686	I	Open-label, multicenter study	No study results posted as study is ongoing
	Ranosidenib	NCT04762602	I	Open label single-arm study	No study results posted as study is ongoing
HER2	Pertuzumab + Trastuzumab	NCT02091141	II	Non-randomized, multicenter, open-label, multiple basket study	ORR 23%, PFS 4 months, OS 10.9 months
	Trastuzumab Deruxtecan	NCCH1805	II	Multicenter, single-arm study	ORR 36.4%, PFS 5.1 months, OS 7.1 months
		NCT04482309	II	Multicenter, multi-cohort, open-label study	ORR 56.3%, PFS 7.4 months
	Trastuzumab + FOLFOX	NCT04722133	II	Investigator-initiated, open-label, non-randomized, single-arm, multi-institutional study	ORR 29.4%, PFS 5.1 months, OS 10.7 months
	Neratinib	NCT01953926	II	Single-arm, multi-cohort, basket study	ORR 16%, PFS 1.4 months, OS 5.4 months
	Zanidatamab	NCT04466891	IIb	Global, multicenter, open- label, single-arm study	ORR 41.3%, PFS 5.5 months, OS 15.5 months
	Trastuzumab + Tucatinib	NCT04579380	II	Multi-cohort, open-label, international study	ORR 46.7%, PFS 5.5 months
BRAF	Dabrafenib + Trametinib	NCT02034110	II	Open-label, non-randomized basket study	ORR 47%, PFS 9 months, OS 13.5 months
	Binimetinib + Encorafenib	NCT03839342	II	Single-center, open-label study	No study results posted as study is ongoing
KRAS	Adagrasib	NCT03785249	I/II	Multicohort study	ORR 50%, PFS 11.3 months, OS 15.1 months
	MRTX1133	NCT05737706	I/II	Open-label, non-randomized first-in-human study	No study results posted as study was terminated
	BI-3 700 674	NCT06056024	I	Open-label dose-finding, non-randomized study	No study results posted as study is ongoing
	Divarasib + Cetuximab	NCT04449874	Ia/Ib	Dose-escalation and dose-expansion study	ORR 62.5%, PFS 8.1 months
	Glecirasib	NCT05002270	I/II	Multicenter, open-label study	No study results posted as study is ongoing
NTRK	Larotrectinib	NCT02122913	I	Open-label, randomized study	ORR 92%, no patients discontinued treatment due to adverse events
		NCT02637687	I/II	Open-label, non-randomized study	ORR 79%, PFS 28.3 months, OS 44.4 months
		NCT02576431	II	Multicenter, open-label study	No study results posted as study is ongoing
	Entrectinib	NCT02097810	I	Single-arm, open-label, first-in-human study	ORR 64.5%, PFS 20.8 months
		NCT02568267	II	Open-label, multicenter, global basket study	ORR 57%, PFS 11.2 months, OS 21 months
RET	Pralsetinib	NCT03037385	I/II	Multi-cohort, open-label study	ORR 57%, PFS 7.4 months, OS 13.6 months
	Selpercatinib	NCT03157128	I/II	Open-label, basket study	ORR 43.9%, PFS 13.2 months, OS 18 months

### FGFR2 fusions

FGFR signaling plays a crucial role in numerous physiological processes, including the regulation of cell proliferation, growth, and survival, which makes it a vulnerable target for oncogenic exploitation [123]. FGFR2 gene fusions and other rearrangements—initially identified in CCAs in 2013—have since been detected in ∼13%–14% of iCCA [86, 87, 124], representing some of the most therapeutically actionable genetic alterations in this malignancy. Recent studies have further validated FGFR2 fusions in iCCA using next-generation sequencing, fluorescence *in situ* hybridization, and immunohistochemistry, providing comprehensive molecular and pathological confirmation [47]. The first two targeted therapies approved for CCA treatment were pemigatinib and infigratinib, both ATP-competitive, reversible FGFR inhibitors. These oral agents showed overall response rates (ORRs) of 37% and 23.1%, respectively, in phase II single-arm clinical trials that enrolled patients with advanced CCA harboring FGFR2 fusions or rearrangements (Table 2) [125, 126], leading to Food and Drug Administration (FDA) Accelerated Approval in April 2020 and May 2021. Nonetheless, a recent trial reported that the most common treatment-emergent adverse events of pemigatinib were hyperphosphatemia, alopecia, and diarrhea, with 10.2% of patients discontinuing treatment due to adverse events, most frequently intestinal obstruction and acute kidney injury [127].

A third selective FGFR inhibitor, futibatinib, forms a covalent and irreversible bond with the P-loop cysteine residue in the FGFR kinase domain. In a phase II trial involving patients with previously treated advanced FGFR2-rearranged iCCA, futibatinib achieved an ORR of 41.7%, with a median response duration of 9.7 months [128]. These promising results led to FDA Accelerated Approval for this indication in September 2022. Another orally available selective pan-FGFR kinase inhibitor with clinical efficacy in FGFR-altered solid tumors is erdafitinib (JNJ-42756493), which has already been approved for the treatment of adult patients with locally advanced or metastatic urothelial carcinoma carrying susceptible FGFR3 mutations, who have progressed after at least one line of systemic therapy [129]. In an international, single-arm phase II trial (NCT04083976), erdafitinib demonstrated notable antitumor activity in patients with advanced solid tumors, including CCA-harboring FGFR alterations (fusions or mutations), achieving an ORR of 52% and a disease control rate (DCR) of 97% in the CCA subgroup [130, 131]. In addition, findings from the open-label, single-arm phase IIa LUC2001 trial (NCT02699606) confirmed the efficacy of erdafitinib in Asian patients with FGFR-altered advanced CCA, reporting an investigator-assessed ORR of 40.9%, with a median progression-free survival (PFS) of 5.6 months and OS of 40.2 months [132]. A recent study revealed that FGFR2 signaling drives NF-κB–dependent glycolysis in iCCA, and that treatment with pemigatinib, infigratinib, or futibatinib can effectively reprogram tumor metabolism, thereby conferring a novel targetable vulnerability in iCCA [133].

Additional next-generation inhibitors are being developed to address resistance to prior FGFR-targeted therapies, including the FGFR2-selective inhibitor tinengotinib, the pan-FGFR kinase inhibitor derazantinib, and gunagratinib [134–136]. Tinengotinib demonstrates promising efficacy and manageable safety in FGFR2-altered CCA, including cases resistant to prior FGFR inhibitors. A phase III trial is underway to further assess its clinical benefits compared to standard treatments (NCT05948475). Derazantinib is currently being evaluated in the phase II study (NCT03230318) for its efficacy in patients with FGFR-fused iCCA who experienced disease progression following at least one line of standard chemotherapy, demonstrating a DCR of 74.8% and a median PFS of 5.7 months. Interim analyses from the same study showed a DCR of 79% in cohort 2, which included patients with FGFR-mutated or -amplified iCCA. Notably, derazantinib exhibited clinically relevant anti-tumor activity across all patients, regardless of the FGFR alteration type [134, 137].

### IDH1 mutations

The R132 mutation in IDH1 leads to a gain-of-function neomorphic activity that converts α-ketoglutarate into 2-hydroxyglutarate, an oncometabolite known to inhibit histone and DNA demethylases, thereby inducing widespread epigenetic alterations and contributing to tumorigenesis [138]. This type of mutation has been reported in ∼13%–25% of patients with iCCA [139]. Ivosidenib, an orally available small-molecule inhibitor targeting mutant IDH1, received FDA approval in August 2021 for the treatment of IDH1-mutant CCA that is refractory to chemotherapy. This decision was based on a phase III clinical trial that demonstrated a significant improvement in PFS compared to placebo [140, 141]. Although OS did not reach statistical significance, this outcome may have been affected by the fact that 70% of patients in the placebo arm crossed over to ivosidenib after disease progression [140]. Nevertheless, much work remains to be done to improve the prognosis of patients with IDH-mutant CCA [138]. Other IDH1 inhibitors currently under clinical investigation in CCA patients include olutasidenib (NCT03684811), crelosidenib (NCT04521686), and ranosidenib (NCT04762602). Considering that IDH1 mutations can impair the repair of double-stranded DNA breaks [142, 143], several clinical trials of poly(ADP-ribose) polymerase inhibitors are enrolling patients with IDH1-mutated CCA, such as olaparib (NCT03212274), olaparib in combination with the ATR inhibitor ceralasertib (NCT03878095), and olaparib plus durvalumab (NCT03991832).

### HER2 mutations

HER2, a member of the ErbB family of RTKs, is overexpressed more frequently in eCCA (17.4%) and gallbladder cancers (19.1%) than in iCCA (4.8%) [144]. In the phase IIa MyPathway basket trial, a dual-antibody regimen consisting of pertuzumab and trastuzumab produced an ORR of 23% and a median PFS of 4.0 months in 39 patients with HER2-amplified and/or overexpressing CCA [145], leading to its inclusion in the NCCN guidelines as a recommended option for previously treated HER2-positive CCA. Trastuzumab deruxtecan, an antibody–drug conjugate, achieved an ORR of 36.4%, including two complete responses, a median PFS of 4.0 months, and a median OS of 10.9 months in a similar phase II cohort (*n* = 22) [146]. Notably, a partial response was also observed in one out of eight patients with HER2-low expressing disease. This agent is currently being evaluated in an ongoing phase II trial involving patients with CCA (NCT04482309). Additionally, a separate phase II study (NCT04722133) assessed the efficacy of trastuzumab in combination with FOLFOX chemotherapy (folinic acid, 5-fluorouracil, and oxaliplatin) as a second-line treatment in 34 HER2-positive CCA patients, showing an ORR of 29.4%, a median PFS of 5.1 months, and a median OS of 10.7 months [147] (Table 2).

The oral, irreversible pan-ErbB tyrosine kinase inhibitor neratinib has also been assessed in patients with previously treated HER2-mutant CCA, which differs from HER2-positive disease [148]. In the phase II CCA cohort involving 25 evaluable patients—including 11 with CCA—neratinib achieved an ORR of 16%, with a median PFS of 2.8 months and a median OS of 5.4 months. Among the CCA subgroup, the median PFS and OS were 1.4 months and 5.4 months, respectively [148].

Zanidatamab, a novel bispecific antibody that targets two HER2 epitopes similarly to trastuzumab and pertuzumab, reported an interim ORR of 41.3% and a median OS of 15.5 months in a phase I trial enrolling 17 patients with advanced HER2-positive CCA [149]. This agent is undergoing further evaluation in clinical trials enrolling patients with CCA and other CCA subtypes (NCT04466891 and NCT03929666). In addition, ongoing studies are investigating alternative HER2-targeted strategies for HER2-overexpressing CCA, including the combination of trastuzumab and the HER2-directed tyrosine kinase inhibitor tucatinib (NCT04579380), as well as novel antibody–drug conjugates such as A166 (NCT03602079) and zanidatamab zovodotin (NCT03821233).

### BRAF mutations

The incidence of mutations in the serine/threonine kinase BRAF varies considerably depending on the anatomical location of the CCA, with the V600E variant being the most prevalent form. This mutation is detected in ∼5% of patients with iCCA [150, 151]. In the phase II, multicenter basket trial (NCT02034110), the combination of dabrafenib (a BRAF inhibitor) and trametinib (a MEK inhibitor) was tested in patients with advanced CCA harboring the BRAF V600E mutation who had progressed following standard therapies, including 39 cases of iCCA. This regimen achieved an ORR of 47%. The median PFS and OS were reported as 9.0 months and 13.5 months, respectively, highlighting the clinical potential of dual BRAF and MEK inhibition in this subset of patients [152, 153]. In addition, a separate phase II trial (NCT03839342) is currently underway to evaluate the efficacy and safety of the combination of binimetinib and encorafenib in adult patients with advanced solid tumors harboring non-V600E BRAF mutations who have failed prior standard treatments (Table 2).

### KRAS mutations

The KRAS oncogene, part of the RAS gene family, encodes a small GTPase that normally facilitates the conversion of GTP into GDP. Mutations in KRAS impair this hydrolytic activity, locking KRAS in a persistently active GTP-bound state. This sustained activation promotes unchecked cell proliferation and contributes to oncogenesis in CCA and various other cancers [154]. Most KRAS mutations in CCA are single-base missense substitutions, with G12D, G12C, and G12V being the most common variants. Although effective targeted therapies for KRAS mutations in CCA have historically been scarce, recent innovations in drug development have begun to overcome the challenge of KRAS being considered “undruggable.” The phase II trial (NCT03785249) investigated the KRAS G12C-specific inhibitor adagrasib (MRTX849) in 64 patients with advanced solid tumors harboring this mutation, including 8 with CCA. Among the CCA subgroup, the confirmed ORR was 50%, with median PFS and OS of 11.3 months and 15.1 months, respectively [155]. Additional KRAS G12C inhibitors are currently under clinical evaluation (NCT04449874, NCT05002270) [156, 157].

Furthermore, non-covalent selective inhibitors specifically targeting the KRAS G12D mutation are being explored in clinical trials. One such agent, MRTX1133, is currently undergoing evaluation in a phase I study involving patients with advanced solid tumors (NCT05737706) [158]. In parallel, the development of pan-KRAS inhibitors is advancing. BI-2865, a novel pan-KRAS inhibitor, demonstrates activity against a broad spectrum of KRAS mutations—including G12C/D/F/V, G13C/D, and V14I—potentially covering up to 95% of KRAS-driven cancers, thus holding substantial therapeutic promise. Another compound with similar characteristics, BI-3700674, is planned to enter a phase I clinical trial (NCT06056024) [159].

The long-standing belief that KRAS is an “undruggable” target has been challenged by the recent development of a range of KRAS mutation-specific inhibitors. These advances offer promising new therapeutic avenues for managing KRAS-driven subtypes of CCA. However, a major hurdle in the continued progress of KRAS-targeted therapies is the emergence of resistance, which limits both the effectiveness and sustainability of treatment responses [160].

### Neurotrophic tropomyosin receptor kinase fusions

Gene fusions involving the neurotrophic tropomyosin receptor kinase (NTRK) represent a rare but actionable genetic event, occurring in ∼0.2% of CCAs [161]. The selective NTRK inhibitors larotrectinib and entrectinib have demonstrated significant antitumor activity across multiple phase I/II studies, yielding ORRs between 57% and 79% in patients with NTRK fusion-positive malignancies, with complete responses reported in 7% to 16% of cases [162, 163]. Among the enrolled CCA patients, partial responses were observed in two out of three individuals. These promising results led to the FDA approval of both agents for the treatment of metastatic or unresectable solid tumors harboring NTRK fusions.

### RET fusions

RET fusions are also rare in CCAs, with reported frequencies of 0.15% in iCCA and 0.11% in eCCA [164]. In a phase I/II trial (NCT03037385), selective RET inhibitor pralsetinib demonstrated broad antitumor activity in RET fusion-positive solid tumors excluding thyroid and non-small cell lung cancers. The trial reported an ORR of 57%, with a median PFS of 7 months and OS of 14 months [165]. Similarly, another phase I/II open-label basket study (NCT03157128), assessed the RET inhibitor selpercatinib in patients with RET fusion-positive solid tumors aside from lung or thyroid origins. The ORR reached 44%, with median PFS and OS of 13.2 and 18 months, respectively [166]. These findings supported the FDA's accelerated approval of selpercatinib in September 2022 for adults with advanced or metastatic RET fusion-positive solid tumors who had previously received systemic therapy or lacked satisfactory treatment options [167].

## Conclusion and perspectives

Approximately 40% of patients diagnosed with advanced CCA harbor identifiable genomic alterations—such as FGFR2 fusions, IDH1 mutations, and HER2 amplifications—that are potentially actionable, providing critical insights for the implementation of precision-targeted therapies [140, 168, 169]. The recent FDA approvals of FGFR inhibitors (pemigatinib, infigratinib, futibatinib), IDH1 inhibitors (ivosidenib), and HER2-targeted regimens (e.g. trastuzumab deruxtecan) further validate the clinical feasibility of molecularly guided treatment strategies [141]. Given these developments, multiple expert consensus guidelines now advocate for routine comprehensive molecular profiling in advanced CCA. Looking ahead, clinical trials assessing targeted agents should increasingly employ stratified trial designs or focus on biomarker-defined patient populations to improve therapeutic precision. The promising clinical outcomes observed with FGFR and HER2 inhibitors further support their consideration for first-line therapeutic application.

Despite recent U.S. FDA approvals of multiple targeted agents for CCA, the clinical application of these therapies continues to face major hurdles [170–172]. A significant challenge stems from tumor heterogeneity—different tumor subclones within a patient may harbor distinct molecular changes—leading to variability in patient responses to the same targeted agents [173–175]. Importantly, acquired resistance to targeted therapies (e.g. FGFR/IDH inhibitors) commonly emerges within months, significantly undermining long-term therapeutic efficacy. Consequently, future research should focus on the following strategic directions: (i) integration of multi-omics platforms (genomic/epigenetic/transcriptomic) to refine molecular classification and identify novel vulnerabilities; (ii) developing next-generation inhibitors (e.g. tinengotinib for FGFR resistance) and rational combination regimens (e.g. PARP inhibitors + ivosidenib for IDH1-mutant CCA); (iii) targeting non-genetic mechanisms, such as epigenetic reprogramming (HDAC/SIRT inhibitors) and ncRNA-mediated pathways (e.g. exosomal LINC01812); and (iv) investigating stromal crosstalk (CAF-mediated signaling, TAM-driven YAP activation) to counteract microenvironment-driven resistance. [85, 176, 177].

In conclusion, molecularly guided targeted therapy has emerged as a promising approach in the management of CCA [178]. Future clinical studies should aim to refine and expand the utility of these therapies, potentially establishing new standards of care. Alongside this, innovative clinical trial models are urgently needed to optimize patient outcomes, reduce treatment costs, and accelerate the development of novel therapeutics. The discovery and validation of predictive biomarkers for targeted therapies remain in an early phase [170, 179–181]. There is a critical need for biomarkers that can anticipate both treatment efficacy and potential adverse effects. In addition, hepatocyte transplantation is also a promising approach for alleviating liver failure [182]. Advancing our understanding of the molecular landscape of CCA will be essential for broadening the clinical arsenal and ultimately enhancing survival and quality of life for patients with advanced disease.
